# Supplementary material for: Antiviral and Anti-Inflammatory Activities of Fluoxetine in a SARS-CoV-2 Infection Mouse Model
Source: Int J Mol Sci. 2022 Nov 7;23(21):13623. doi: 10.3390/ijms232113623 (PMC9657171; doi:10.3390/ijms232113623)
Supplement: Supplementary file 1 [file ijms-23-13623-s001.zip › ijms-1880590-supplementary.pdf]

Supplemental information for

Antiviral and anti-inflammatory activities of Fluoxetine in a SARS-CoV-2 infection mouse model

Running title: Fluoxetine in a SARS-CoV-2 infection mouse model

David Péricat[1*], Stephen Adonai Leon-Icaza[1*], Marina Sanchez Rico[2,3,4], , Christiane Mühle[5], Iulia Zoicas[5], Fabian Schumacher[6], Rémi Planès[1], Raoul Mazars[1], Germain Gros[1], Alexander Carpinteiro[7], Katrin Anne Becker[7], Jacques Izopet[8,9] , Olivier Neyrolles[1], Burkhard Kleuser[6], Frédéric Limosin[2,3,4], Erich Gulbins[7], Johannes Kornhuber[5], Etienne Meunier[1], Nicolas Hoertel[2,3,4,*], Céline Cougoule[1,*#]

[1] Institute of Pharmacology and Structural Biology (IPBS), University of Toulouse, CNRS, Toulouse; France
[2] Université de Paris Cité, Paris, France.
[3] Assistance Publique-Hôpitaux de Paris (AP-HP), Hôpital Corentin-Celton, DMU Psychiatrie et Addictologie, Hôpital Corentin-Celton, Département de Psychiatrie et d'Addictologie de l'adulte et du sujet âgé, Issy-les-Moulineaux, 92130, France.
[4] INSERM, Institut de Psychiatrie et Neurosciences de Paris (IPNP), UMR_S1266, Paris, France.
[5] Department of Psychiatry and Psychotherapy, University Hospital, Friedrich-Alexander-University of Erlangen-Nuremberg, Erlangen, Germany.
[6] Freie Universität Berlin, Institute of Pharmacy, Königin-Luise-Str. 2+4, 14195 Berlin, Germany
[7] Institute for Molecular Biology, University Medicine Essen, University of Duisburg-Essen, Essen, Germany.
[8] Infinity, Université Toulouse, CNRS, INSERM, UPS, 31300 Toulouse, France.
[9] Laboratoire de Virologie, CHU Toulouse, Hôpital Purpan, 31300 Toulouse, France.

* these authors contributed equally

Corresponding author#
Céline Cougoule, Ph.D., H.D.R.,
IPBS-Toulouse, CNRS-Université Toulouse III 205 Route de Narbonne - 31077 Toulouse cedex 4, France
Phone: +33-(0)5 61 17 54 61
Email: celine.cougoule@ipbs.fr

Supplementary Materials and Methods

Ethic statements: The collection of patient data and tissue for human airway organoid generation was performed according to the guidelines of the European Network of Research Ethics Committees following European and national law. The responsible accredited ethical committees reviewed and approved this study in accordance with the Medical Research Involving Human Subjects Act. The CHU of Toulouse and CNRS approved protocol CHU 19 244 C and Ref CNRS 205782. All patients participating in this study consented to scientific use of their material; patients can withdraw their consent at any time, leading to the prompt disposal of their tissue and any derived material. Non-tumor lung tissue from two independent donors receiving surgical treatment for lung cancer were used to derive human airway organoids.

SARS-CoV-2 virus production

The BetaCoV/France/IDF0372/2020 isolate was supplied by Sylvie van der Werf and the National Reference Centre for Respiratory Viruses hosted by Institute Pasteur (Paris, France). The mNeonGreen (mNG) reporter SARS-CoV-2 were based on 2019-nCoV/USA_WA1/2020 isolated from the first reported SARS-CoV-2 case in the USA, and provided through the World Reference Center for Emerging Viruses and Arboviruses (WRCEVA), and UTMB investigator, Dr. Pei Yong Shi. The following reagents were deposited by Centers for Disease Control and Prevention and obtained through BEI Resources, NIAID, NIH: SARS-Related Coronavirus 2, Isolate USA/CA_CDC_5574/2020, NR-54011, SARS-Related Coronavirus 2, Isolate hCoV-19/Japan/TY7-503/2021 (Brazil P.1), NR-54982, contributed by National Institute of Infectious Diseases. SARS-CoV-2 Delta and Omicron BA-5 variants were isolated and provided by Pr. J. Izopet from the Toulouse hospital, France. SARS-CoV-2 isolates were amplified by infecting Vero E6 cells (ATCC CRL-1586) (MOI 0.005) in DMEM (Gibco) supplemented with 10mM HEPES and 1% penicillin-streptomycin (Gibco). The supernatant was harvested at 48 h post-infection when cytopathic effects were observed, cell debris were removed by centrifugation,

and aliquots were frozen at −80°C. Viral stocks were titrated by $TCID_{50}$ assays in Vero E6 cells. Typical titers were 5 to 10× 10^6 PFU/ml.

Cell death and viability

Cell death was measured by quantification of the lactate dehydrogenase (LDH) release into the cell supernatant using LDH Cytotoxicity Detection Kit (Takara). Briefly, 50 µL cell supernatant were incubated with 50 µL LDH substrate and incubated for 15 min. The enzymatic reaction was stopped by adding 50 µL of stop solution. Maximal cell death was determined with whole cell lysates from unstimulated cells incubated with 1% Triton X-100. Cell viability was measured by quantification of intracellular ATP using CellTiter-Glo® One Solution Assay (Promega) according to manufacturer's instructions.

Mice

C57BL/6 mice (Charles River, Sulzfeld, Germany; 22 - 26g) and 32 K18-hACE2 mice (Jackson Laboratory-USA, 18 - 20g) were housed in individually ventilated cages under standard laboratory conditions (12:12 light:dark cycle, lights on at 07:00 h, 22 °C, 60% humidity, food and water ad libitum) in groups of 4 mice per cage.

Quantitative real-time PCR analysis of viral RNA and inflammatory genes

Lung tissue was first homogenized in 3 ml PBS in an M tube using a GentleMACS dissociator (Miltenyi biotec). For each mouse, 150 µl of lung tissue homogenate were mixed with 1 mL TRIzol Reagent (Invitrogen) and stored at -80 0C at least 48 h before to be taken out the BSL-3 facility. Total RNA was extracted using the RNeasy mini kit (Qiagen) and reverse-transcribed (150ng) with the High-Capacity cDNA Reverse Transcription Kit (Thermo Fisher Scientific). Gene expression levels of cytokines and chemokines were assessed with the SYBR Select Master Mix (Thermo Scientific) and relative quantifications were determined using the 2^- Ct method with β-actin as reference. Viral loads were performed as previously described.41,42 Briefly, viral load quantifications were carried out by linear regression employing a standard

curve of 6 known quantities of plasmids containing the RdRp sequence (107 to 100 copies) and qPCRs were performed in TaqMan Universal PCR Master Mix (Thermo Fisher Scientific). qPCRs for gene expression and viral load were performed in triplicate and assessed with an ABI 7500 real-time PCR system (Applied Biosystems). Primers and probe sequences are provided in Supplemental Table S1.

Supplementary Figures

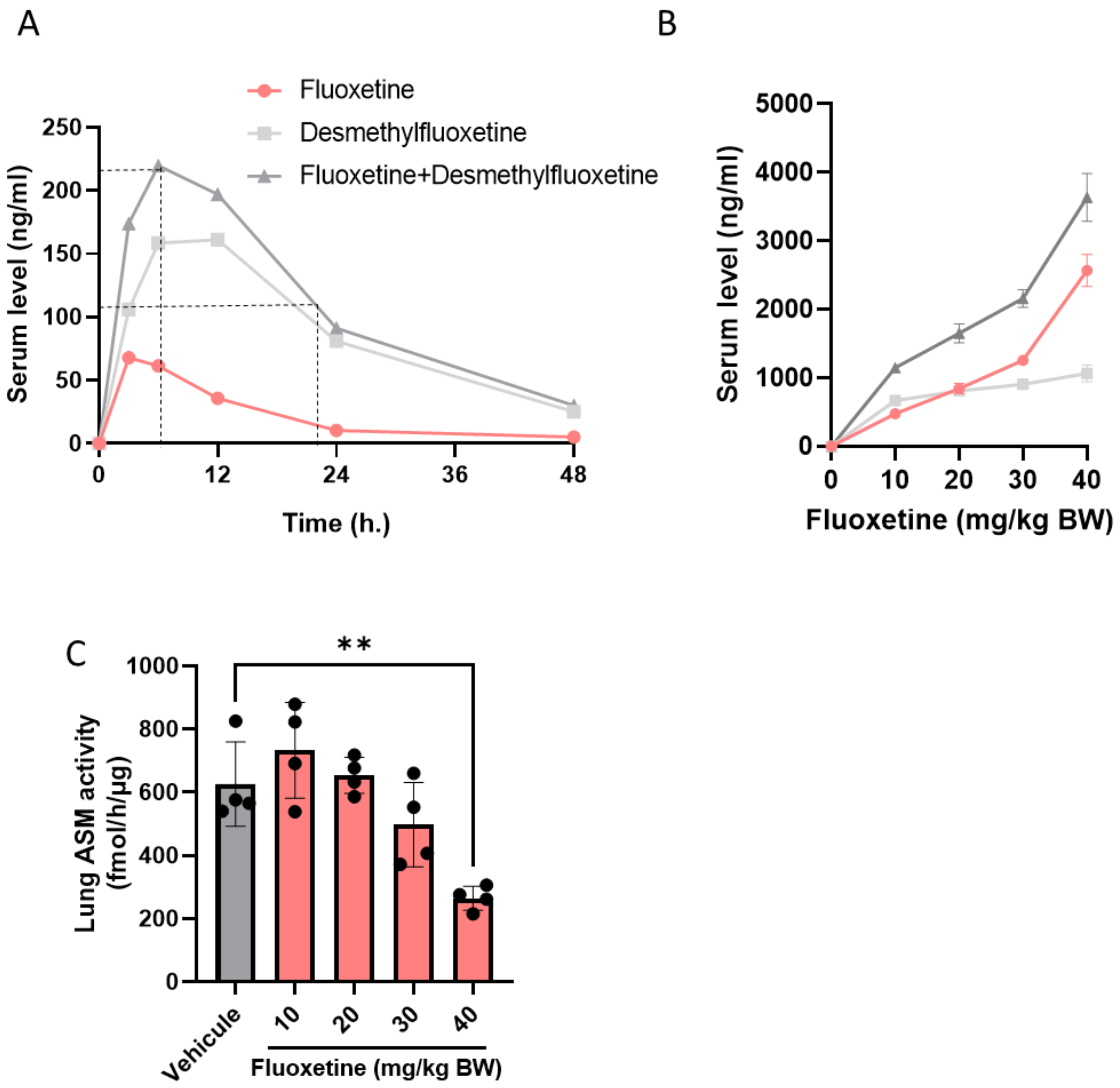

Supplemental Figure S1 (related to Figure 1): Pharmacokinetic studies of fluoxetine in C57BL/6 mice. (A) 5 mg/kg fluoxetine was applied per oral gavage. Serum levels of fluoxetine and its active metabolite desmethylfluoxetine were measured over time, i.e., after 3, 6, 12, 24 and 48 h. (B)(C) Mice were injected intraperitoneally with saline (0.9% NaCl solution) or several doses of fluoxetine (i.e., 10, 20, 30 and 40 mg/kg) in a volume of 10 ml/kg (n = 4 mice/group) and blood and lung tissue were collected 6 h later. Fluoxetine and desmethylfluoxetine was measured in serum (B) and ASM activity was measured in the lung tissue (C); ** $p < 0.01$.

A

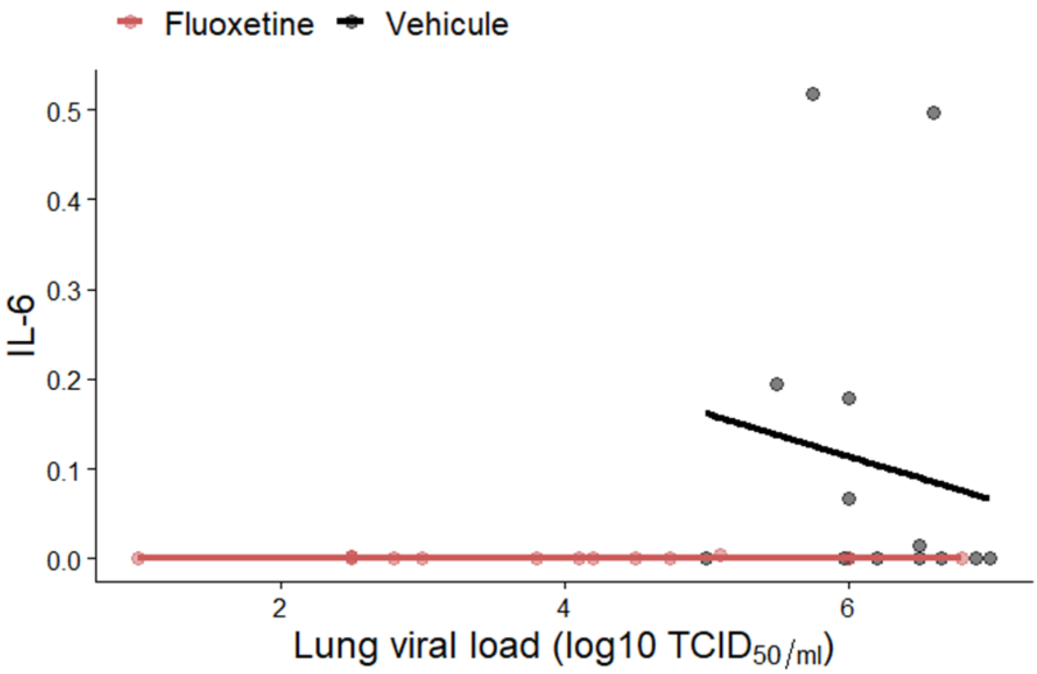

B

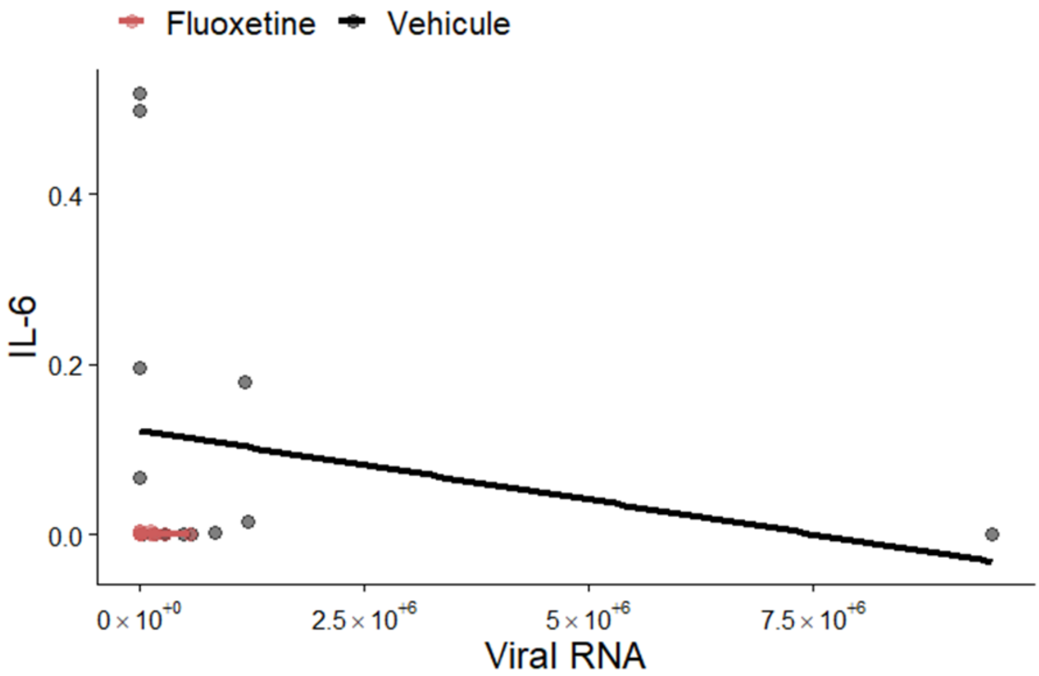

Supplemental Figure S2 (related to Figure 1). Association between viral load and IL-6 expression in mice at day 2 and 6 post-infection combined. (A) association of IL-6 expression level with Lung $TCID_{50}$ in saline- and fluoxetine-treated mice; (B) association of IL-6 expression level with viral RNA load in saline- and fluoxetine-treated mice.

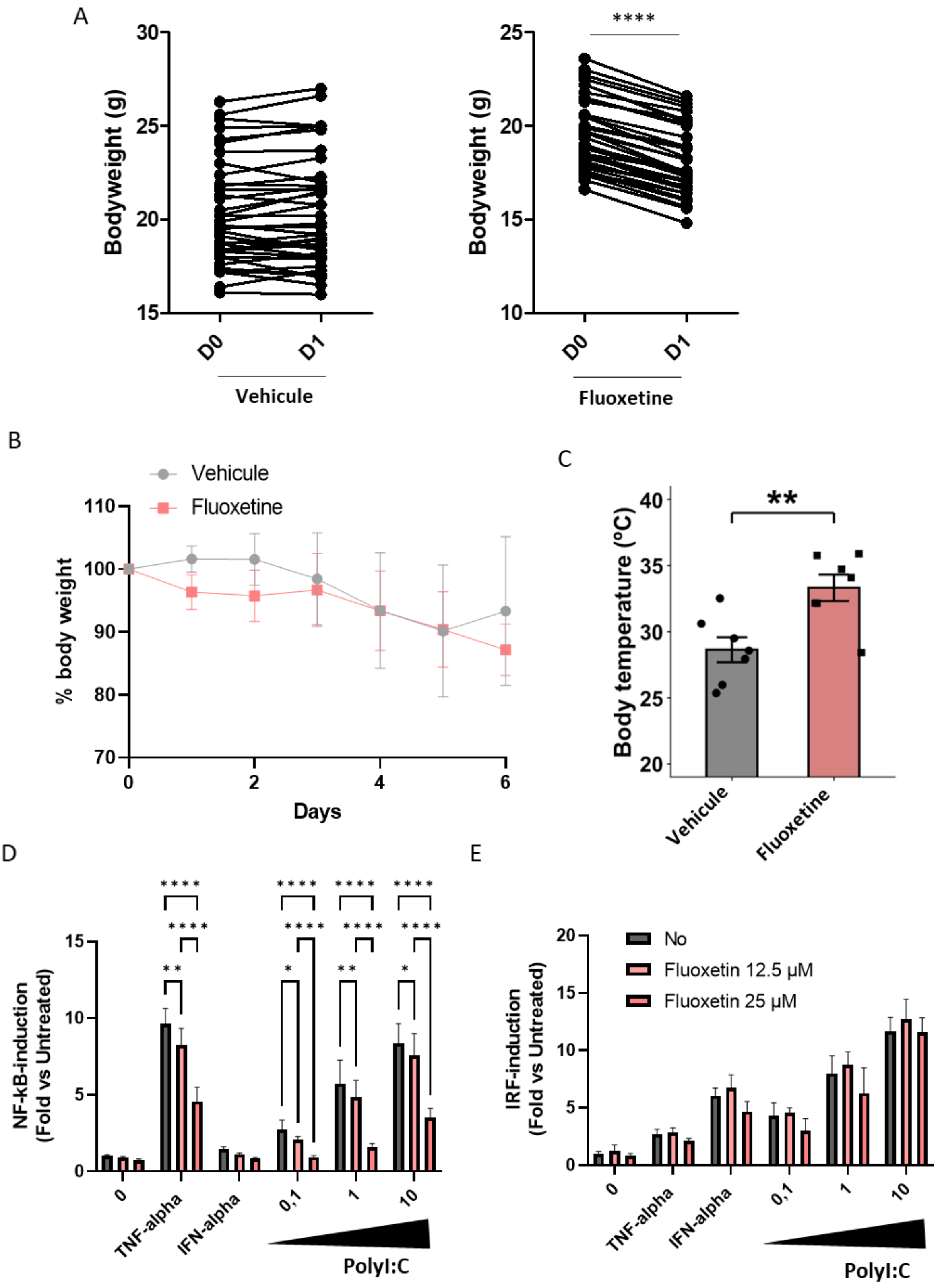

Supplemental Figure S3 (related to Figure 1): Fluoxetine effect on body weight and temperature (A) Body weight of K18-hACE2 mice after the first treatment with saline or fluoxetine; (B) Body weight was recorded daily and the mean percentage weight change from

normalized initial weight was plotted (mean ± SEM); (C) body temperature recorded at day 6 post-infection. (D-E) A549-dual A549 cells were incubated with fluoxetine (12.5-25 μM) for 1h prior to be treated with various activators of the NF-kB and/or IRF pathways including TNF-α (10 ng/mL), IFN-α (10 ng/mL), and poly I:C (10 – 0.1 μg/mL). fold increase of NF-kB (D) and IRF (E) pathway activation are shown. Fold increase of NF-kB, IRF pathways activation and percentage of cell viability were calculated compared to the untreated condition. Data show one experiment representative of two performed independently. *, p<0.05, **, p<0.01; ****, p<0.0001.

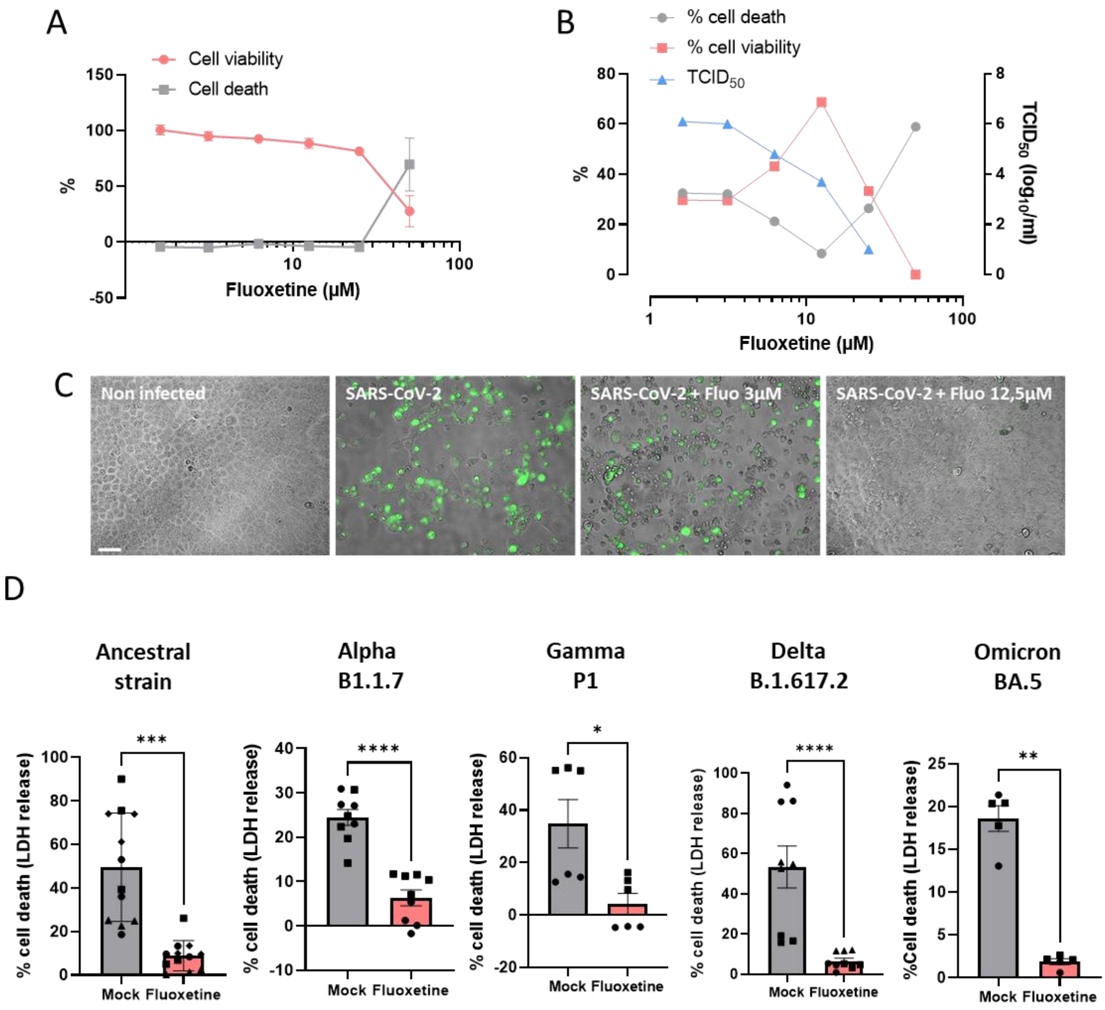

Supplemental Figure S4 (related to Figure 3): Fluoxetine activity *in vitro*. Vero E6 cells were treated with fluoxetine at 1,.5 to 50 μM for 1 hr prior infection and then infected with SARS-CoV-2 for 72 h. (A) Fluoxetine cytotoxicity on Vero E6 cells measured 72 h post-treatment;

(B) Dose-response analysis of SARS-CoV-2 replication, cell viability and cell death with fluoxetine treatment. (C) Representative images 72 h post infection of Vero E6 cells infected or not with mNG-reporter SARS-CoV-2 strain, treated or not with fluoxetine. Scale bar = 100 µm. (D) Percentage of cell death measured by LDH release assay on Vero E6 cells infected with SARS-CoV-2 variants with or without fluoxetine treatment (12.5µM) for 72h. Data represent men ± SEM from two independent experiments performed in triplicates. *, p<0.05; **, p<0.01; ***, p<0.001; ****, p<0.0001.

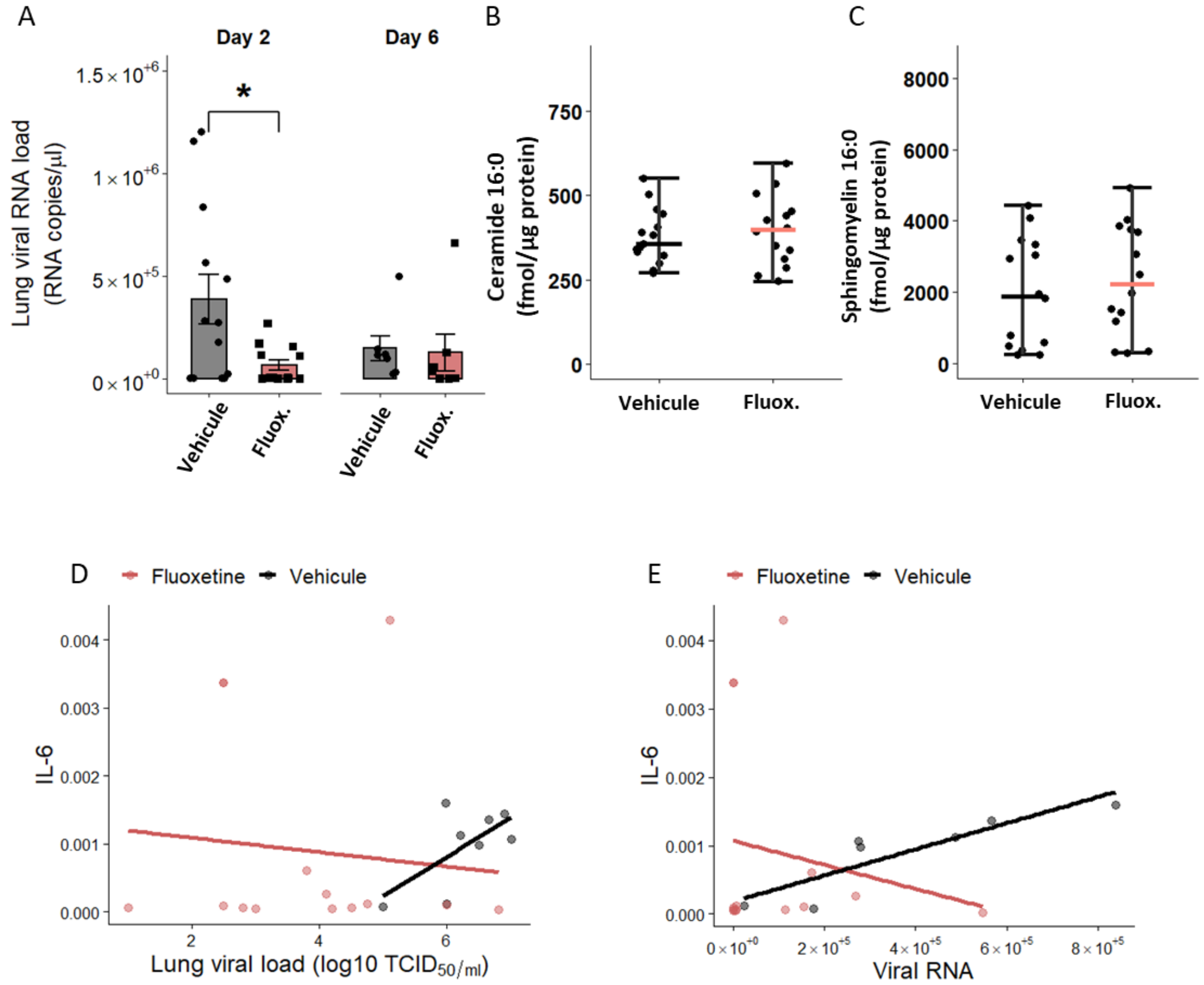

Supplemental Figure S5. Data after excluding outliers. (A) Viral RNA levels in the lungs of saline- and fluoxetine–treated mice infected with SARS-CoV-2 at day 2 and 6 post-infection expressed as Log_{10} SARS-CoV-2 RNA genome copies after excluding outliers; P<0.05 (B) Fluoxetine effect on ceramide 16:0 after excluding outliers; (C) Fluoxetine effect on

sphingomyelin 16:0 after excluding outliers; (D) association of IL-6 expression level with Lung $TCID_{50}$ in saline- and fluoxetine-treated mice after excluding outliers; (E) association of IL-6 expression level with viral RNA load in saline- and fluoxetine-treated mice after excluding outliers.

Supplemental Table S1 List of qPCR primers

Target	Primers 5’-3’	Reference
**Cytokines and chemokines**		
**IL-6**	F- TACCACTTCACAAGTCGGAGGC<br>R- CTGCAAGTGCATCATCGTTGTTC	OriGene
**CXCL10**	F- ATTGCCACGATGAAAAAGAATGAT<br>R- AGACCAAGGGCAATTAGGACTAGC	Shimizu K, et al. *J Immunol*. 2011;186(10):5927-5937. doi:10.4049/jimmunol.1003351
**TNFα**	F- GGTGCCTATGTCTCAGCCTCTT<br>R- GCCATAGAACTGATGAGAGGGAG	OriGene
**CCL2**	F- GCTACAAGAGGATCACCAGCAG<br>R- GTCTGGACCCATTCCTTCTTGG	OriGene
**IL-1β**	F- TGGACCTTCCAGGATGAGGACA<br>R- GTTCATCTCGGAGCCTGTAGTG	OriGene
**β-actin**	F- GCTGTGCTGTCCCTGTATGCCTCT<br>R- CCTCTCAGCTGTGGTGGTGAAGC	Koblansky AA, et al. *Immunity*. 2013;38(1):119-130. doi:10.1016/j.immuni.2012.09.016
**Viral load**		
**RdRp**	F- GTGARATGGTCATGTGTGGCGG<br>R- CAAATGTTAAAAACACTATTAGCATA<br>Probe-<br>FAM- CAGGTGGAACCTCATCAGGAGATGC –<br>TAMRA	Corman VM, et al. *Euro Surveill*. 2020;25(3):2000045. doi:10.2807/1560-7917.ES.2020.25.3.2000045<br><br>Rebendenne A, et al. *J Virol*. 2021;95(8):e02415-20. doi:10.1128/JVI.02415-20

Supplemental Table S2. Associations of fluoxetine *versus* saline exposure with inflammatory markers.

	Day 2		Day 6	
	Fluoxetine vs. Saline	Fluoxetine vs. Saline	Fluoxetine vs. Saline	Fluoxetine vs. Saline
	Viral load	Viral RNA	Viral load	Viral RNA
	OR [95% CI]	OR [95% CI]	OR [95% CI]	OR [95% CI]
**IL6**				
Crude	0.47 (0.23 - 0.94; 0.042)*	0.47 (0.23 - 0.94; 0.042)*	0.28 (0.12 - 0.65; 0.013)*	0.28 (0.12 - 0.65; 0.013)*
Adjusted	0.43 (0.15 - 1.19; 0.115)	0.42 (0.20 - 0.86; 0.025)*	0.48 (0.15 - 1.59; 0.259)	0.28 (0.11 - 0.67; 0.018)*
**TNFα**				
Crude	0.32 (0.17 - 0.58; 0.001)*	0.32 (0.17 - 0.58; 0.001)*	0.34 (0.14 - 0.85; 0.040)*	0.34 (0.14 - 0.85; 0.040)*
Adjusted	0.31 (0.12 - 0.76; 0.017)*	0.41 (0.24 - 0.69; 0.003)*	1.08 (0.39 - 3.00; 0.888)	0.35 (0.14 - 0.87; 0.045)*
**CXCL10**				
Crude	0.44 (0.22 - 0.88; 0.028)*	0.44 (0.22 - 0.88; 0.028)*	0.39 (0.15 - 1.02; 0.080)	0.39 (0.15 - 1.02; 0.080)
Adjusted	0.42 (0.15 - 1.16; 0.107)	0.53 (0.27 - 1.04; 0.076)	0.75 (0.19 - 2.89; 0.681)	0.40 (0.15 - 1.07; 0.095)
**CCL2**				
Crude	0.26 (0.15 - 0.45; <0.001)*	0.26 (0.15 - 0.45; <0.001)*	0.48 (0.17 - 1.30; 0.174)	0.48 (0.17 - 1.30; 0.174)
Adjusted	0.37 (0.17 - 0.81; 0.020)*	0.29 (0.17 - 0.51; <0.001)*	1.01 (0.25 - 4.12; 0.984)	0.49 (0.18 - 1.35; 0.194)

* Two-sided p-value is significant (p<0.05).

Supplementary Table S3. List of reagents used for ALI staining

Antibody	Cat N°	Supplier	Dilution
Anti acetylated Tubulin	Sc-23950	Santa Cruz	1/300
Goat anti Mouse IgG Dylight 550	GTX MU003D550NHS	Immunoreagent	1/1000
Alexa Fluor 647 Phalloidin	A22287	Invitrogen	1/40
